# Prevalence of HIV Preexposure Prophylaxis Prescribing Among Persons With Commercial Insurance and Likely Injection Drug Use

**DOI:** 10.1001/jamanetworkopen.2022.21346

**Published:** 2022-07-12

**Authors:** Carl G. Streed, Jake R. Morgan, Mam Jarra Gai, Marc R. Larochelle, Michael K. Paasche-Orlow, Jessica L. Taylor

**Affiliations:** 1 Section of General Internal Medicine, Department of Medicine, Boston University School of Medicine and Boston Medical Center, Massachusetts; 2 Center for Transgender Medicine and Surgery, Boston Medical Center, Massachusetts; 3 Department of Health Law, Policy, and Management, Boston University School of Public Health, Massachusetts; 4 Grayken Center for Addiction, Boston Medical Center, Massachusetts

## Abstract

**Importance:**

Although HIV preexposure prophylaxis (PrEP) implementation among persons who inject drugs has been inadequate, national HIV monitoring programs do not include data on PrEP, and specific trends in PrEP use are not well understood.

**Objective:**

To estimate HIV PrEP uptake among commercially insured persons with opioid or stimulant use disorder by injection drug use (IDU) status.

**Design, Setting, and Participants:**

This cross-sectional study used deidentified data from the MarketScan Commercial Claims and Encounters Database to identify a sample of 547 709 commercially insured persons without HIV but with opioid and/or stimulant use disorder, including 110 592 with evidence of IDU between January 1, 2010, and December 31, 2019. Data were analyzed from November 1, 2020, to July 1, 2021.

**Exposures:**

Persons with opioid and/or stimulant use disorder and evidence of IDU were identified through claims data.

**Main Outcomes and Measures:**

The outcome was receipt of tenofovir disoproxil fumarate and emtricitabine for PrEP as identified from filled pharmacy claims. Multivariable logistic regression was used to assess the association of demographic and clinical characteristics with receipt of PrEP.

**Results:**

The study cohort included 211 609 (28.6%) females and 336 100 (61.4%) males with a combined mean (SD) age of 34.8 (13.1) years, including 110 592 individuals with evidence of IDU. During the study period, 508 (0.09%) persons with opioid and/or stimulant use disorder, including 170 (0.15%) with evidence of IDU, received PrEP. Receipt of PrEP increased from 0.001 to 0.243 per 100 person-years from 2010 through 2019 among the entire cohort and from 0.000 to 0.295 per 100 person-years among those with IDU. In multivariable analysis, PrEP use was more likely among males (adjusted odds ratio [aOR] 8.72; 95% CI, 6.39-11.89), persons with evidence of IDU (aOR, 1.47; 95% CI, 1.21-1.79), and persons with evidence of sexual risk indications for PrEP (aOR, 23.68; 95% CI, 19.57-28.66).

**Conclusions and Relevance:**

In this cross-sectional study of commercially insured persons with opioid and/or stimulant use disorder, HIV PrEP delivery remained low, including among those with evidence of IDU. PrEP should be consistently offered alongside substance use disorder treatment and other harm reduction and HIV prevention services.

## Key Points

**Question:**

What is the prevalence of HIV preexposure prophylaxis (PrEP) among commercially insured persons with opioid and/or stimulant use disorder by injection drug use (IDU) status?

**Findings:**

In this cross-sectional study of 547 709 commercially insured persons with opioid and/or stimulant use disorder, including 110 592 persons with evidence of IDU, 0.09% of the population overall and 0.15% of those with evidence of IDU received a PrEP prescription.

**Meaning:**

In this study, HIV PrEP delivery for persons with substance use disorder, including those who inject drugs, remained low.

## Teaser

This cross-sectional study estimated uptake of HIV preexposure prophylaxis (PrEP) among commercially insured persons with substance use disorder by injection drug use status.

## Introduction

Public health progress in reducing the incidence of HIV among people who inject drugs (PWID) in the US has been impeded by the opioid crisis. Short-acting, illicitly manufactured fentanyl analogs and the ubiquity of concurrent stimulant use increase injection frequency and have contributed to injection-related HIV outbreaks in at least 11 states.^1^ Preexposure prophylaxis (PrEP) is a potent HIV prevention strategy among PWID, reducing HIV incidence by 74% in those with detectable medication levels and by 49% overall.^2^ Daily tenofovir disoproxil fumarate and emtricitabine (TDF/FTC) as PrEP is therefore recommended for PWID who have shared injection equipment or engaged in sexual risk behaviors, including condomless sex with partners of unknown HIV status, in the previous 6 months.^3,4^

Available estimates of HIV risk behaviors among PWID suggest that PrEP should be widely implemented in this population.^5,6^ However, PrEP uptake by PWID remains low,^7,8^ despite a growing body of data associated with successful delivery to those experiencing homelessness and with psychosocial vulnerabilities.^9,10^ Although PrEP implementation among US PWID has been inadequate, national HIV monitoring programs do not include data on PrEP, and specific trends in PrEP use are not well understood.

A recent analysis of a national database of commercially insured persons (MarketScan Commercial Claims and Encounters Database; IBM) identified PWID aged 16 years or older who were prescribed TDF/FTC for PrEP between 2010 and 2014.^11^ The researchers found a very low but significantly increasing trend in the proportion of persons prescribed TDF/FTC for PrEP during the study period. Although they evaluated basic demographic information, including administrative sex (male or female) and age, indications for HIV PrEP were not explored or reported. To inform future interventions to increase PrEP uptake among PWID, it is essential to understand PrEP use and trends in this specific population, whose needs may vary greatly from those with sexual risk behaviors alone.

Accordingly, the goal of this study was to use MarketScan to estimate PrEP uptake on a per-year basis from 2010 to 2019 among commercially insured persons in the US with opioid and/or stimulant use disorder with and without evidence of injection drug use (IDU). We stratified by evidence of IDU, defined as evidence of infectious complications indicating likely injection, hypothesizing that uptake would be higher in persons with evidence of IDU. We assessed temporal trends in receipt of TDF/FTC for PrEP and examined characteristics of persons receiving 1 or more TDF/FTC prescriptions for PrEP during the study period.

## Methods

### Data

In this cross-sectional study, we identified a cohort of persons aged 16 years or older with opioid or stimulant use disorder included in the MarketScan database from January 1, 2010, to December 31, 2019. MarketScan is an insurance claims–based data set that includes ambulatory and inpatient visits, laboratory and diagnostic testing, and outpatient pharmacy claims from a large, geographically diverse convenience sample of the US commercially insured population.^12^ Because the MarketScan data were deidentified, the Boston University Medical Campus Institutional Review Board deemed this research exempt from review and the requirement for informed consent. The study followed the Strengthening the Reporting of Observational Studies in Epidemiology (STROBE) guidelines for reporting observational studies.

### Cohort Definition

The cohort included persons aged 16 years or older with opioid use disorder, those with stimulant use disorder, and those with co-occurring opioid and stimulant use disorder. Individuals could be entered into the data set in any year from 2010 through 2019 and were followed for as long as they were included in the data set or until the end of the study period on December 31, 2019. We excluded individuals with evidence of HIV or hepatitis B virus on the basis of diagnosis codes and the presence of non-PrEP antiretroviral treatment. No other inclusion or exclusion criteria were used.

We identified opioid use disorder by using an algorithm by Wakeman et al.^13^ Briefly, the algorithm defines opioid use disorder as 1 or more inpatient or 2 or more outpatient claims within 3 months for opioid dependence that did not occur during a long-term opioid prescribing episode (ie, for chronic pain) or as 1 or more inpatient or outpatient claims for opioid dependence, abuse, or use accompanied by a confirmatory diagnosis or procedure, such as evidence of opioid overdose, injection-related infection, detoxification, or medication treatment; the full algorithm is presented as eFigure 1 in Wakeman et al.^13^ We adapted this algorithm for stimulants by requiring either 1 or more inpatient or 2 or more outpatient claims within 3 months of each other for stimulant dependence or 1 or more inpatient or outpatient claims for stimulant dependence, abuse, or use and a confirmatory diagnosis (eg, stimulant overdose), stimulant-related inpatient care, or evidence of injection-related infections.

### Outcome

Our outcome of interest was receipt of TDF/FTC for HIV PrEP. We identified receipt of PrEP through pharmacy claims for TDF/FTC with a duration of at least 28 days and without concurrent prescription of a third agent, which, in persons without HIV infection, is consistent with postexposure prophylaxis. During the study period, TDF/FTC was the only US Food and Drug Administration (FDA)–approved PrEP formulation for persons with injection-related HIV risk; thus, we did not include prescriptions for tenofovir alafenamide and emtricitabine, which were FDA approved for PrEP on October 3, 2019, for persons at risk for HIV transmission through anal sex.

### Exposure Measures

We included several demographic and clinical variables that we hypothesized would be associated with PrEP prescription. First, we assessed demographic characteristics, including patient age (continuous); sex (categorical); and relationship to the primary enrollee (categorical), for example, whether the patient was the covered employee, a spouse, or a dependent. This latter category was evaluated because of variability in the privacy protections afforded to nonprimary enrollees for PrEP-related services during the study period and data revealing that concerns about inadvertent disclosure to primary enrollees was a barrier to PrEP uptake among young persons on their parents’ health insurance plans.^14^ MarketScan does not include data on race and ethnicity. Next, we included clinical characteristics, including evidence of IDU (dichotomous),^13^ evidence of sexual risk behaviors (dichotomous, a potential PrEP indication), and receipt of an FDA-approved medication for opioid use disorder (MOUD; categorical). Medication for opioid use disorder was defined by receipt of buprenorphine, naltrexone, or methadone on the basis of pharmacy claims or in-office administration.^15^ Evidence of IDU was defined by the diagnosis of injection-related infections (eg, hepatitis C virus, soft tissue infection, infective endocarditis, infective arthritis).^13^ Sexual risk was measured as a dichotomous variable defined by diagnosis codes for high-risk behavior or evidence of a sexually transmitted infection (eTable in the Supplement). Finally, we included the calendar year (categorical) of a person’s first enrollment in the data set to characterize changing PrEP prescribing trends over time and to control for time trends in regression analyses.

### Statistical Analysis

We summarized each variable of interest in the population overall and stratified between persons with and without evidence of IDU. We first compared the characteristics using a χ^2^ test. Next, we conducted a multivariable analysis to assess the association between demographic and clinical factors and the receipt of a PrEP prescription. We adjusted for the variables (ie, age, sex, enrollment time, evidence of IDU, evidence of sexual risk behaviors, substance use disorder diagnosis, receipt of MOUD, and enrollment year) and calculated the adjusted odds ratios (aORs) and 95% CIs, measuring the odds of receiving a PrEP prescription associated with each study variable. Analyses were conducted from November 1, 2020, to July 1, 2021, using SAS, version 9.4 (SAS Institute Inc). Significance was set at *P* < .05 using a 2-tailed test.

## Results

We identified 547 709 persons with an opioid and/or stimulant use disorder, including 110 592 (20.2%) with evidence of IDU (hereafter referred to as PWID). The mean (SD) time enrolled in the cohort was 3.94 (2.64) years. Across all cohort characteristics, PWID differed from persons without evidence of IDU. Of note, PWID were older (mean [SD] age, 36.33 [13.91] vs 34.37 [12.84] years; *P* < .001), were less likely male (female, 62 092 [56.15%]; male, 274 008 [62.69%]; *P* < .001), had a longer enrollment period (mean [SD], 3.94 [2.64] vs 2.76 [2.27] years; *P* < .001), and were less likely to receive MOUD (32 072 [29.00%] vs 143 903 [32.92%]; *P* < .001) compared with those without evidence of IDU.

Of 508 persons who received PrEP, 338 (0.08%) had no evidence of IDU and 170 (0.15%) were PWID (Table 1). Among persons with a pharmacy claim for PrEP, PWID were more likely to be female (26 [15.29%]) vs 19 [5.62%]; *P* < .001) and had a longer enrollment period (4.72 vs 3.56 years; *P* < .001) compared with persons with no evidence of IDU (Table 2). After adjusting for individual characteristics and substance use diagnoses, having a pharmacy claim for TDF/FTC was associated with being male (aOR, 8.72; 95% CI, 6.39-11.89), having evidence of IDU (aOR, 1.47; 95% CI, 1.21-1.79), and having evidence of sexual risk behavior (aOR, 23.68; 95% CI, 19.57-28.66) (Table 3). The incidence of having a pharmacy claim for TDF/FTC increased from 0.001 per 100 person-years in 2010 to 0.243 per 100 person-years in 2019 in the overall cohort. Among persons with evidence of IDU, the rate of PrEP prescribing increased from 0 in 2010 (no PrEP prescriptions among PWID in the data set in 2010) to 0.295 in 2019, exceeding the rate of prescribing in the overall cohort (Figure).

**Table 1.** Patient Characteristics of Persons With Opioid and/or Stimulant Use Disorder Stratified by Evidence of IDU, 2010-2019

Characteristic	No. (%)			P value
	No evidence of IDU	Evidence of IDU	Total	
Patients	437 117 (79.81)	110 592 (20.19)	547 709 (100)	NA
PrEP receipt				
No	436 779 (99.92)	110 422 (99.85)	547 201 (99.91)	<.001
Yes	338 (0.08)	170 (0.15)	508 (0.09)	
Age, mean (SD), y	34.37 (12.84)	36.33 (13.91)	34.76 (13.09)	<.001
Enrollment time, mean (SD), y	2.76 (2.27)	3.94 (2.64)	3.00 (2.40)	<.001
Year of initial enrollment				
2010	137 499 (31.46)	43 346 (39.19)	180 845 (33.02)	<.001
2011	66 337 (15.18)	15 386 (13.91)	81 723 (14.92)	
2012	45 753 (10.47)	10 353 (9.36)	56 106 (10.24)	
2013	53 362 (12.21)	13 881 (12.55)	67 243 (12.28)	
2014	42 776 (9.79)	8872 (8.02)	51 648 (9.43)	
2015	22 154 (5.07)	5466 (4.94)	27 630 (5.04)	
2016	18 284 (4.18)	4575 (4.14)	22 859 (4.17)	
2017	19 280 (4.41)	4101 (3.71)	23 381 (4.27)	
2018	21 774 (4.98)	3543 (3.20)	25 317 (4.62)	
2019	9898 (2.26)	1069 (0.97)	10 967 (2.00)	
Sex				
Female	163 109 (37.31)	48 500 (43.85)	211 609 (38.64)	<.001
Male	274 008 (62.69)	62 092 (56.15)	336 100 (61.36)	
Region				
Northeast	102 247 (23.39)	22 928 (20.73)	125 175 (22.85)	<.001
North central	86 948 (19.89)	20 069 (18.15)	107 017 (19.54)	
South	168 472 (38.54)	46 529 (42.07)	215 001 (39.25)	
West	73 285 (16.77)	19 722 (17.83)	93 007 (16.98)	
Unknown	6165 (1.41)	1344 (1.22)	7509 (1.37)	
Relationship to primary enrollee				
Employee	212 455 (48.60)	47 129 (42.62)	259 584 (47.39)	<.001
Spouse	95 670 (21.89)	29 590 (26.76)	125 260 (22.87)	
Child/other	128 992 (29.51)	33 873 (30.63)	162 865 (29.74)	
Sexual risk behavior				<.001
No	423 736 (96.94)	104 545 (94.53)	528 281 (96.45)	
Yes	13 381 (3.06)	6047 (5.64)	19 428 (3.55)	
Received MOUD				
No	293 214 (67.08)	78 520 (71.00)	371 734 (67.87)	<.001
Yes	143 903 (32.92)	32 072 (29.00)	175 975 (32.13)	
MOUD type				
No MOUD	293 214 (67.08)	78 520 (71.00)	371 734 (67.87)	<.001
Buprenorphine or naloxone	110 702 (25.33)	22 262 (20.13)	132 964 (24.28)	
Oral naltrexone	12 508 (2.86)	3106 (2.81)	15 614 (2.85)	
Injectable naltrexone	4402 (1.01)	1074 (0.97)	5476 (1.00)	
Injectable buprenorphine	369 (0.08)	101 (0.09)	470 (0.09)	
Methadone	5255 (1.20)	962 (0.87)	6217 (1.14)	
≥2 MOUDs	10 667 (2.44)	4567 (4.13)	15 234 (2.78)	
Plan type				
POS	33 107 (7.57)	8065 (7.29)	41 172 (7.52)	<.001
PPO	269 314 (61.61)	68 472 (61.91)	337 786 (61.67)	
HMO	43 212 (9.89)	11 924 (10.78)	55 136 (10.07)	
Other	91 484 (20.93)	22 131 (20.01)	113 615 (20.74)	
SUD diagnosis				
OUD	391 752 (89.62)	98 402 (87.16)	500 635 (86.19)	<.001
Cocaine	7427 (1.70)	4018 (3.63)	11 445 (2.09)	
Other stimulants	5084 (1.16)	3582 (3.24)	8666 (1.58)	
OUD and other stimulants	4451 (1.02)	1626 (1.47)	6077 (1.11)	
OUD and cocaine	6341 (1.45)	1861 (1.68)	8202 (1.50)	
Other stimulants and cocaine	6372 (1.46)	1352 (1.22)	7724 (1.41)	
OUD, other stimulants, and cocaine	15 690 (3.59)	1873 (1.69)	17 563 (3.21)	

Abbreviations: HMO, health maintenance organization; IDU, injection drug use; MOUD, medication for opioid use disorder; NA, not applicable; OUD, opioid use disorder; POS, point of service; PPO, preferred provider organization; PrEP, preexposure prophylaxis; SUD, substance use disorder.

**Table 2.** Patient Characteristics of Persons Who Received a PrEP Prescription by Evidence of IDU, 2010-2019

Characteristic	No. (%)			P value
	No evidence of IDU	Evidence of IDU	Total	
Patients	338 (66.54)	170 (33.46)	508 (100)	NA
Age, mean (SD), y	33.58 (11.01)	32.18 (11.95)	33.12 (11.34)	.15
Enrollment time, mean (SD), y	3.56 (2.77)	4.72 (2.80)	3.94 (2.83)	<.001
Year of initial enrollment				
2010	59 (17.64)	37 (21.76)	96 (18.90)	<.001
2011	15 (4.44)	15 (8.82)	30 (5.91)	
2012	18 (5.33)	17 (3.35)	35 (6.89)	
2013	25 (7.40)	25 (14.71)	50 (9.84)	
2014	35 (10.36)	11 (2.17)	46 (9.06)	
2015	35 (10.26)	14 (8.24)	49 (9.65)	
2016	42 (12.43)	17 (10.00)	59 (11.61)	
2017	34 (10.06)	20 (11.76)	54 (10.63)	
2018	52 (15.38)	7 (4.12)	59 (11.61)	
2019	23 (6.80)	7 (4.12)	30 (5.91)	
Sex				
Female	19 (5.62)	26 (15.29)	45 (8.86)	<.001
Male	319 (94.38)	144 (84.71)	463 (91.14)	
Region				
Northeast	92 (27.22)	40 (23.53)	132 (25.98)	.71
North central	50 (14.79)	29 (17.06)	79 (15.55)	
South	109 (32.25)	50 (29.41)	159 (31.30)	
West	86 (25.44)	50 (29.41)	136 (26.77)	
Unknown	1 (0.30)	1 (0.59)	2 (0.39)	
Relationship to primary enrollee				
Employee	220 (65.09)	94 (55.29)	314 (61.81)	.008
Spouse	31 (9.17)	10 (5.88)	41 (8.07)	
Child/other	87 (25.74)	66 (38.82)	153 (30.12)	
Sexual risk behavior				
No	174 (51.48)	79 (46.47)	253 (49.80)	.29
Yes	164 (48.52)	91 (53.53)	255 (50.20)	
Received MOUD				
No	219 (64.79)	106 (62.35)	325 (63.98)	.59
Yes	119 (35.21)	64 (37.65)	183 (36.02)	
MOUD type				
No MOUD	219 (67.39)	106 (62.35)	325 (63.98)	.61
Buprenorphine or naloxone	46 (13.61)	26 (15.29)	72 (14.17)	
Oral naltrexone	41 (12.13)	17 (10.00)	58 (11.42)	
Injectable naltrexone	10 (2.96)	4 (2.35)	14 (2.76)	
Injectable buprenorphine	0 (0.0)	0(0.00)	0 (0.0)	
Methadone	1 (0.30	0 (0.00)	1 (0.20)	
≥2 MOUDs	21 (6.21)	17 (10.00)	38 (7.48)	
Plan type				
POS	49 (14.50)	20 (11.76)	69 (13.58)	.51
PPO	159 (47.04)	82 (48.24)	241 (47.44)	
HMO	33 (9.76)	23 (13.53)	56 (11.02)	
Other	97 (28.70)	45 (26.47)	142 (27.95)	
SUD diagnosis				
OUD	207 (61.24)	94 (55.29)	301 (59.25)	.49
Cocaine	12 (3.55)	11 (6.47)	23 (4.53)	
Other stimulants	41 (12.13)	35 (20.59)	76 (14.96)	
OUD and other stimulants	9 (2.66)	7 (4.12)	16 (3.15)	
OUD and cocaine	5 (1.48)	3 (1.76)	8 (1.57)	
Other stimulants and cocaine	49 (14.50)	16 (9.41)	65 (12.80)	
OUD, other stimulants, and cocaine	15 (4.44)	4 (3.35)	19 (3.74)	

Abbreviations: HMO, health maintenance organization; IDU, injection drug use; MOUD, medication for opioid use disorder; NA, not applicable; OUD, opioid use disorder; POS, point of service; PPO, preferred provider organization; PrEP, preexposure prophylaxis; SUD, substance use disorder.

**Table 3.** Association of Patient Characteristics With Receipt of Preexposure Prophylaxis Prescription Among 547 709 Persons With Opioid and/or Stimulant Use Disorder

Characteristic	aOR (95% CI)
Age, y	
<30	1 [Reference]
≥30	0.78 (0.60-1.00)
Sex	
Female	1 [Reference]
Male	8.72 (6.39-11.89)
Enrollment time, y	1.35 (1.29-1.42)
IDU	
No	1 [Reference]
Yes	1.47 (1.21-1.79)
Sexual risk behavior	
No	1 [Reference]
Yes	23.68 (19.57-28.66)
SUD diagnosis	
OUD	1 [Reference]
Other stimulants	6.16 (4.66-8.15)
Cocaine	1.61 (1.04-2.50)
Other stimulants and cocaine	5.31 (3.95-7.14)
OUD and other stimulants	3.28 (1.96-5.51)
OUD and cocaine	1.29 (0.63-2.61)
OUD, other stimulants, and cocaine	2.29 (1.43-3.69)
Relationship to primary enrollee	
Employee	1 [Reference]
Spouse	0.41 (0.29-0.57)
Child/other	0.40 (0.30-0.53)
Received MOUD	
No	1 [Reference]
Yes	1.17 (0.96-1.43)
Year of initial enrollment	
2010	1 [Reference]
2011	1.42 (0.92-2.17)
2012	2.30 (1.52-3.47)
2013	2.21 (1.53-3.20)
2014	3.98 (2.66-5.97)
2015	6.78 (4.49-10.24)
2016	9.68 (6.39-14.68)
2017	10.02 (6.42-15.62)
2018	15.28 (9.71-24.04)
2019	24.69 (14.33-42.53)

Abbreviations: aOR, adjusted odds ratio; IDU, injection drug use; MOUD, medication for opioid use disorder; OUD, opioid use disorder; SUD, substance use disorder.

**Figure.**
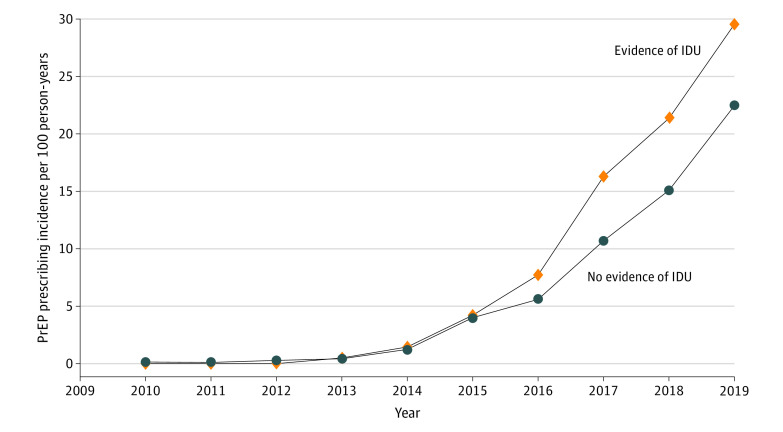
Preexposure Prophylaxis (PrEP) Prescribing in Persons With Opioid and/or Stimulant Use Disorder IDU indicates injection drug use.

## Discussion

HIV PrEP has been approved for use since 2012 and has been endorsed since 2013 by the Centers for Disease Control and Prevention and FDA as an effective prevention strategy to reduce HIV incidence in PWID. PrEP is an essential tool for medical and public health systems across the country confronting increased HIV transmission in this population.^16,17^ However, our results reveal that PrEP prescribing to commercially insured PWID and other persons with substance use disorders remains low. We found that fewer than 1 in 500 PWID had pharmacy claims for PrEP. Furthermore, in the cohort overall, just 1.7% of those with sexual risk behavior (a more widely addressed indication for PrEP) had pharmacy claims for PrEP. These findings suggest that this evidence-based prevention strategy is massively underused.

Although disappointing, our finding that outpatient pharmacy-filled prescriptions for HIV PrEP are rare among commercially insured patients with opioid and/or stimulant use disorder is consistent with previous literature on general PrEP prescribing rates.^11^ PrEP prescribing in our data is alarmingly low, even among persons with claims data indicating evidence of IDU.

The primary benefit of PrEP is HIV prevention, but the PrEP continuum of care involves a series of touch points that can yield substantial spillover benefits, including behavioral risk reduction support, sexually transmitted infection testing, smoking cessation, and access to other preventive services (eg, vaccination).^18,19,20^ This potential for PrEP to increase overall care engagement is particularly important for those with opioid use disorder given well-documented gaps in preventive care and barriers to initiation of and retention on MOUD in this population.^15,21,22^ Our findings of an association between receipt of MOUD and receipt of PrEP are consistent with previous research revealing that integrating both HIV PrEP and MOUD into care could be an acceptable and beneficial policy, especially in locations where PWID already access services (eg, drug detoxification centers).^23^

Finally, the very low PrEP prescribing rates in our cohort demand interventions to address other well-described PrEP barriers, including the financial burden of medication.^24^ Even very low copays have been shown to discourage use of MOUD,^25^ and existing programs, such as the Health Resources and Services Administration’s Ryan White HIV/AIDS Program, have had great success in mitigating copay burden for HIV retroviral treatment.^26^ Similar interventions that provide PrEP at no out-of-pocket cost may address some cost-related barriers for PWID and others who stand to benefit.^27^

### Limitations

This study has some limitations. Although these data are likely to be accurate for PrEP receipt among patients with a PrEP prescription billed to their commercial insurer, we did not design the study to capture PrEP prescriptions funded through other sources (eg, self-pay, research studies); thus, our findings likely underestimate total PrEP coverage in this population. In addition, these data likely underidentify persons with substance use disorders and PWID because the study design relied on billing in an administrative claims database to identify clinical conditions as proxies of substance use disorder and IDU and did not explicitly identify IDU. Persons with substance use disorders that lead to formal diagnoses and care episodes likely have more severe substance use disorders or injecting behaviors relative to a broader population. Despite the underestimation of the population with substance use disorders and IDU, those identified are likely to benefit most from HIV PrEP because of increased risk behaviors. The methods used also identify a cohort with commercial insurance, at least some contact with the medical system, and medical record data that should readily identify their HIV risk to clinicians. Indeed, these limitations underscore the concerning meaning of our findings, which likely overstate the rate of PrEP among PWID.

Our empirical strategy assumes that the characteristics of our population are not changing dramatically over time in ways that also predict changes in PrEP prescription. However, given the size and national representativeness of commercially insured persons within our data, we did not see dramatic changes over time.

## Conclusions

To achieve the public health goal of reducing new HIV infections by 75% by 2025 and at least 90% by 2030^28^ and to stem the tide of HIV clusters and outbreaks among PWID across the country, no evidence-based prevention strategy can be overlooked. In this cross-sectional study, we found that the rate of PrEP among commercially insured PWID and other persons with opioid and/or stimulant use disorder is very low. Aggressive expansion of PrEP for PWID is urgently needed.

## Notes

Supplement. **eTable.** Diagnostic Codes Utilized
