# Supplementary material for: Prevalence of HIV Preexposure Prophylaxis Prescribing Among Persons With Commercial Insurance and Likely Injection Drug Use
Source: JAMA Netw Open. 2022 Jul 12;5(7):e2221346. doi: 10.1001/jamanetworkopen.2022.21346 (PMC9277489; doi:10.1001/jamanetworkopen.2022.21346)
Supplement: Supplement. — eTable. Diagnostic Codes Utilized [file jamanetwopen-e2221346-s001.pdf]

Supplemental Online Content

Streed CG Jr, Morgan JR, Gai MJ, Larochelle MR, Paasche-Orlow MK, Taylor JL. Prevalence of HIV preexposure prophylaxis prescribing among persons with commercial insurance and likely injection drug use. *JAMA Netw Open*. 2022;5(7):e2221346.
doi:10.1001/jamanetworkopen.2022.21346

eTable. Diagnostic Codes Utilized

This supplemental material has been provided by the authors to give readers additional information about their work.

© 2022 Streed CG Jr et al. *JAMA Network Open.*

eTable 1. Diagnostic Codes Utilized

Text Label	Version	Diagnosis Code
**Substance Use Diagnoses**		
Opioid use disorder	ICD-9	304.0x
		304.7x
	ICD-10	F11x
Cocaine use disorder	ICD-9	304.2x
		305.6x
	ICD-10	F14.2x
		F14.1x
		F14.9x
Other stimulant dependence	ICD-9	304.4x
		305.7x
	ICD-10	F15.2x
		F15.1x
		F15.9x
**Sexual Behavior**		
High-risk sexual behavior	ICD-9	V69.2
High-risk heterosexual behavior	ICD-10	Z72.51
High-risk homosexual behavior	ICD-10	Z72.52
High-risk bisexual behavior	ICD-10	Z72.53
**Sexually transmitted infections**		
Chlamydia	ICD-9	078.88
		079.88
		079.98
		099.41
		099.5
	ICD-10	A74.x
		A56.xx
Gonorrhea	ICD-9	098.xx
	ICD-10	A54.xx
Syphilis	ICD-9	090.xx
		091.xx
		092.xx
		093.xx
		094.xx
		095.xx
		096.xx
		097.xx

© 2022 Streed CG Jr et al. *JAMA Network Open.*

	ICD-10	A50.xx
		A51.0
		A51.49
		A52.3
		A53.0
		A53.9
Herpes simplex	ICD-9	054.1
		054.10
		054.19
	ICD-10	A60.xx
		A53
		A55
		A57
		A58

© 2022 Streed CG Jr et al. *JAMA Network Open.*
